# Sessile volatile drop evaporation under microgravity

**DOI:** 10.1038/s41526-020-00128-2

**Published:** 2020-12-11

**Authors:** Sanjeev Kumar, Marc Medale, Paolo Di Marco, David Brutin

**Affiliations:** 1 Aix-Marseille Universite, CNRS, IUSTI UMR 7343, Marseille, 13013 France; 2 DESTEC, University of Pisa, Largo Lazzarino 1, Pisa, 56122 Italy; 3 Institut Universitaire de France, Paris, 75231 France

**Keywords:** Fluid dynamics, Chemical engineering

## Abstract

The evaporation of sessile drops of various volatile and non-volatile liquids, and their internal flow patterns with or without instabilities have been the subject of many investigations. The current experiment is a preparatory one for a space experiment planned to be installed in the European Drawer Rack 2 (EDR-2) of the International Space Station (ISS), to investigate drop evaporation in weightlessness. In this work, we concentrate on preliminary experimental results for the evaporation of hydrofluoroether (HFE-7100) sessile drops in a sounding rocket that has been performed in the frame of the MASER-14 Sounding Rocket Campaign, providing the science team with the opportunity to test the module and perform the experiment in microgravity for six consecutive minutes. The focus is on the evaporation rate, experimentally observed thermo-capillary instabilities, and the de-pinning process. The experimental results provide evidence for the relationship between thermo-capillary instabilities and the measured critical height of the sessile drop interface. There is also evidence of the effects of microgravity and Earth conditions on the sessile drop evaporation rate, and the shape of the sessile drop interface and its influence on the de-pinning process.

## Introduction

Drops have been fascinating researchers for centuries^1–3^. Topics of interest include water falling onto a hot cooking plate, which is a typical example of Leidenfrost drops^1^, the evaporation of sessile drops with nanoparticle deposition in coffee rings^4^, inkjet printing^5,6^, pesticides sprayed onto leaves^7^, and blood analysis^8,9^. Although sessile drops are simple in geometry, the physics involved in the evaporation process is complex due to the numerous intricate interactions with the substrate and ambient environment, and the fluid nature of the sessile drop itself. An accurate quantitative model of the evaporation process can lead to greater understanding of the evaporation rate and control over the pattern formation or the deposition of particles after the evaporation of a sessile drop. This knowledge can then enhance the efficiency of several applications. The physically rich and complex evaporation of sessile drops is thus of interest to both the academic and industry communities.

Parabolic flight experiments on drops of various fluids have been performed multiple times by The National Centre for Space Studies (CNES), France, and The European Space Agency (ESA) parabolic flight campaigns^10–15^. The existence of thermo-capillary instabilities^14,16^ and the effect of the reduced gravity environment on evaporation^11,17^ and the drop interface^10,18,19^ have already been demonstrated. Parabolic flights have enabled these observations, but such flights are not sufficient in terms of duration or residual acceleration for accurate measurements to be taken. Furthermore, the drop interface is highly sensitive to aircraft vibrations. A better level of microgravity and a longer evaporation time are therefore needed.

**Fig. 1:**
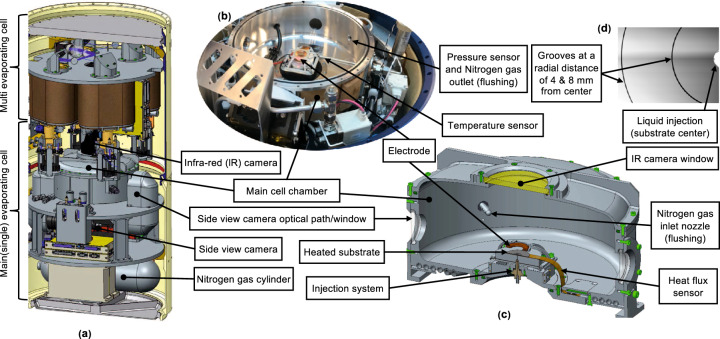
Overview of the ARLES experimental setup. **a** Experiment module on-board the MASER 14 rocket, divided into two parts: the main evaporating and multi-evaporating cells. **b**, **c** Main evaporating cell (MEC) with a detailed schematic. **d** Platinum layered surface crystal silicon wafer substrate (top view) with grooves. Images **a**, **b**, and **c** are credited to the European Space Agency (ESA) and Swedish Space Corporation (SSC).

**Fig. 2:**
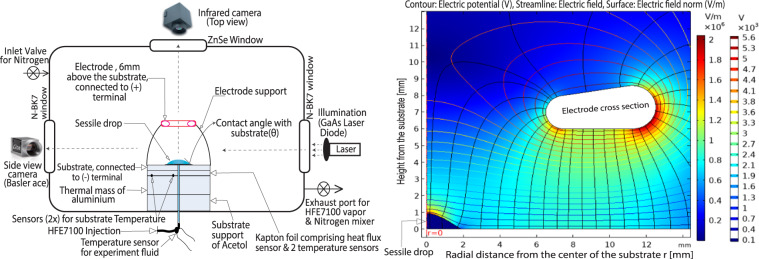
Schematic of the main evaporating cell (MEC) of the ARLES experiment (left). Axisymmetric electric field around sessile drop (right). Readers are advised to refer to web version of this figure for better display.

The Advanced Research on Liquid Evaporation in Space (ARLES) experiment module (see Figs. 1 and 2) was designed to support the investigation of the evaporation process in a controlled environment. ARLES was part of the payload in a SubOrbital Express rocket (MASER 14) and it successfully took place on Monday, 24 June 2019 from the Esrange Space Center in northern Sweden under the collaboration of the ESA and Swedish Space Corporation (SSC). The ARLES experiment was conducted as a preparation for an experiment that is to be performed in the near future at the European Drawer Rack 2 of the International Space Station under the EVAPORATION project of the ESA. The intent is to study evaporating drops of pure fluids as well as drops of fluids that contain a low concentration of metallic nanoparticles. The influence of an electric field is also of interest. The application of an external electrostatic field induces electric stress at the vapor–liquid interface, deforming it and altering the contact angle. The resulting electric forces press the drop against the surface and elongate it in the vertical direction; in addition, electroconvection is induced in the liquid and in the surrounding vapor atmosphere, resulting in a possible enhancement of evaporation rate, which may result useful when gravity-driven convection is suppressed. The scientific objectives include dealing with the flow motion and the thermo-capillary instabilities occurring in the drop, at the drop interface, and in the vapor phase, and investigating the pattern formation on the substrate after the evaporation phase.

ARLES was a collaborative experiment among various teams. Each team focused on different aspects of the experiment to contribute to the overall scientific objectives of the experiment, such as flow motion and thermo-capillary instabilities occurring in the drop, at the drop interface, and in the vapor phase, the pattern formation on the substrate after evaporation of the volatile phase, the deposition of nanoparticles, and the eventual heat transfer enhancement. Our team primarily focused on the analysis of the flow motion and thermo-capillary instabilities occurring in the drop using data from the infrared (IR) (top view) camera and on the evaporation rate and interface evolution of the sessile drop using data from the side-view camera. The experimental results presented here address the effect of microgravity and Earth conditions on the evaporation, thermo-capillary instabilities, drop interface, and de-pinning of a forced sessile drop of hydrofluoroether (HFE-7100) liquid on a heated substrate. The experimental results allow for a comparison of data from both ground and space experiments, thereby providing firm conclusions.

## Results

### Experimental setup and conditions

In Fig. 1a, a complete setup of the ALRES experiment has been shown. It consists of two parts, namely the main evaporating cell (MEC; bottom) and multi-evaporating cells (top). Our current focus is on the MEC experiment. The detailed schematic of the MEC is presented in the Fig. 2 (left) along with its chamber shown in Fig. 1b (top view) and 1c (cut view) with injection system, substrate, and electric field electrode (substrate is connected to the negative (−) terminal and the electrode to the positive (+) terminal). Figure 2 (right) shows the electric field distribution around the sessile drop for the axisymmetric case. For more details, please refer to MEC schematic in Fig. 2.

**Fig. 3:**
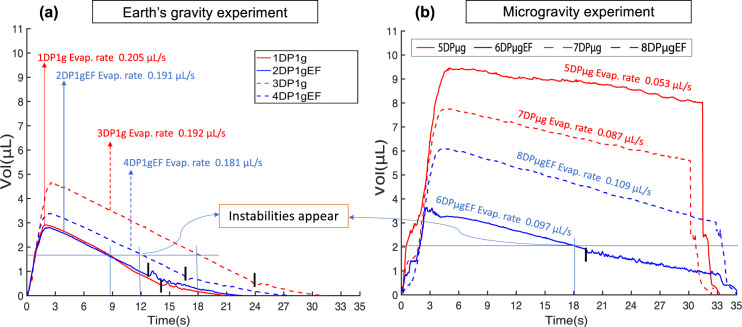
Volume of sessile drops on the heated substrate vs. time. **a** Earth’s gravity. **b** Microgravity conditions. The bar **l** denotes the de-pinning stage of the sessile drops. For the better interpretation, please also refer Table 1 along with figure. Readers are advised to refer to the web version of the figure.

The ideal experimental conditions for the MEC are as follows: target theoretical nominal parameters for microgravity and Earth conditions were set to be similar for the purposes of comparison. The injection velocity of liquid HFE-7100 for sessile drop creation on the heated substrate was 4 μL s^−1^ and the nominal volume of each sessile drop was set at 6 μL. However, multiple ground experiments have shown that it is difficult to precisely control the injection liquid volume with the current injection system and hardware. Even though the actual injected volume of the drops during the ground experiment is lower than the target theoretical nominal value but the actual injected volume of the drops during the microgravity conditions is higher than the target nominal one (see Fig. 3). The temperature of the main test cell was set at 26 °C and the temperature of the substrate was set at 28 °C with an imposed electric field 8 kV for all drops with electric field, except for drop 8DP*μ*gEF under microgravity, for which the field was set at 5.7 kV. Due to the grooves on the substrate, the base diameter of all the sessile drops remained constant (4 mm) during evaporation until the drops de-pinned.

### Experimental results

For Earth gravity, the experimental data from the sensor are as follows: the main cell pressure (inside chamber) *P*_amb_, ambient temperature (inside chamber) *T*_amb_, and substrate center temperature *T*_sc_, and the difference between the substrate center and ambient temperatures (*T*_sc_ − *T*_amb_) were in the ranges 1053–1058 mbar, 26.16–25.87 °C, 27.93–28.00 °C, and 1.84–2.14 °C, respectively, for all drops. Furthermore, the substrate edge temperature *T*_se_ was in the range 28.11–28.24 °C. Thus, (*T*_se_ − *T*_sc_) was in the range 0.16–0.22 °C and (*T*_se_ − *T*_amb_) was in the range 2.04–2.31 °C for all drops.

Similarly, for the microgravity experiment, the sensor data are as follows: the main cell pressure (inside chamber) *P*_amb_, ambient temperature (inside chamber) *T*_amb_, and substrate center temperature *T*_sc_, and the difference between the substrate center and ambient temperatures (*T*_sc_ − *T*_amb_) were in the range 1050–1057 mbar, 25.65–25.21 °C, 27.95–28.08 °C, and 2.39–2.79 °C, respectively, for all drops. The substrate edge temperature *T*_se_ was in the range 28.13–28.18 °C. Thus, (*T*_se_ − *T*_sc_) was in the range 0.15–0.21 °C and (*T*_se_ − *T*_amb_) was in the range 2.58–2.99 °C for all drops. The data and results from the Earth gravity and microgravity experiment are summarized in Table 1.

**Table 1:** Summary of experiments with internal notation.

Drops no.	Drops code	Injected volume (μL)	Evaporation rate during constant contact area (μL s^−1^)			Instabilities appeared at volume	At de-pinning		Volumetric force condition
			Evaporation rate	Uncertainty (plus)	Uncertainty (minus)	(μL)	Volume (μL)	*θ* (deg.)	
1	1DP1g	2.91 at *t* = 2 s	0.205	0.011	0.013	1.6	0.43	7.6	Gravity
2	2DP1gEF	2.81 at *t* = 2.2 s	0.191	0.005	0.007	1.59	0.81	14.6	Gravity with elecrtic field
3	3DP1g	4.67 at *t* = 2.6 s	0.192	0.006	0.004	1.54	0.43	8.6	Gravity
4	4DP1gEF	3.88 at *t* = 2.4 s	0.181	0.012	0.005	1.67	0.76	13.4	Gravity with elecrtic field
5	5DP*μ*g	9.45 at *t* = 5 s	0.053	0.003	0.003	NA	NA	NA	Microgravity
6	6DP*μ*gEF	3.28 at *t* = 4.8 s	0.097	0.014	0.001	2.01	1.83	18.7	Microgravity with electric field
7	7DP*μ*g	7.75 at *t* = 4.6 s	0.087	0.007	0.006	NA	NA	NA	Microgravity
8	8DP*μ*gEF	6.10 at *t* = 4.5 s	0.109	0.009	0.005	NA	NA	NA	Microgravity with electric field
9	9DP*μ*g	6	0.095	NA	NA	NA	NA	NA	Microgravity without electric field (Analytical)

A comparison of the sessile drop volume with respect to time during evaporation is presented in Fig. 3. We can see that in the Earth’s gravity experiment, all drops evaporated from the heated substrate before flushing started, whereas in the microgravity experiment flushing started before evaporation was complete (see the sudden fall in the drop volume). In the latter, only drop 6DP*μ*gEF de-pinned, conversely to Earth’s gravity experiment, where all drops did.

To compare the evaporation rates of sessile drops measured in microgravity experiment, one can refer to the analytical model for evaporation limited by diffusion, first derived by Picknett and Bexon^20^ for a constant contact area (up to de-pinning) and a spherical cap shape. In our experiments, the wetted area between the liquid HFE-7100 and heated substrate was constant with a base diameter of 4 mm (owing to the groove in the substrate). The analytical evaporation rate is thus:

1 $$\frac{dV}{dt}=2\pi {D}_{\mathrm{eff}}{C}_{\mathrm{sat}}LF(\theta )$$

2 $${C}_{\mathrm{sat}}=\frac{{P}_{\mathrm{sat}}{M}_{\mathrm{l}}}{{R}_{\mathrm{gas}}{T}_{\mathrm{amb}}}$$

3 $$F(\theta )=(8.957\ 1{0}^{-5}+0.633\ \theta +0.116\ {\theta }^{2}-0.08878\ {\theta }^{3}+\, 0.01033\ {\theta }^{4})/\sin \ \theta \quad \,{\rm{for}}\quad \pi /18\le \theta \le \pi ,$$

where *L* is the drop base radius, *C*_sat_ is the saturated vapor concentration, *T*_amb_ is the ambient temperature in Kelvin, *R*_gas_ is the universal gas constant, *P*_sat_ is the saturation pressure based on the ambient temperature *T*_amb_ in the MEC, *M*_l_ is the molecular weight of the liquid (HFE-7100), and *D*_eff_ is the diffusion coefficient of HFE-7100 in a nitrogen gas environment. The diffusion coefficient *D*_eff_ was calculated according to the Fuller–Schettler–Giddings equation^21^ and *F*(*θ*) is a function of the contact angle of the sessile drop, derived by Picknett and Bexon^20^.

**Fig. 4:**
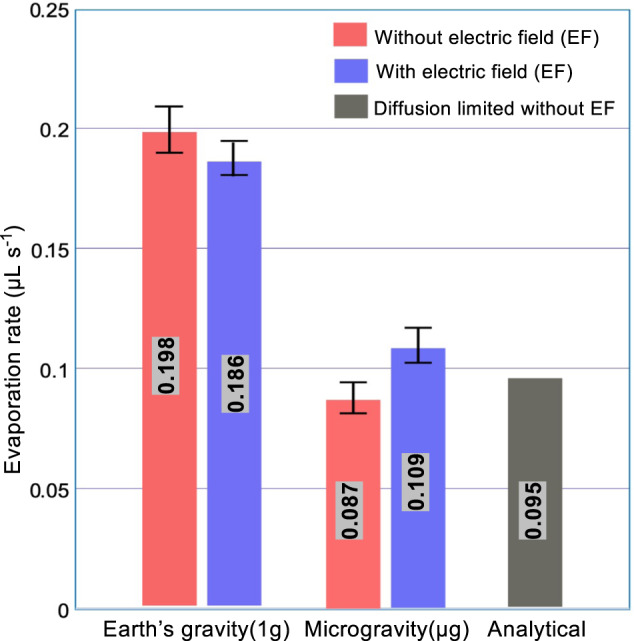
Evaporation rates over the constant contact angle mode. Earth conditions: average evaporation rates of sessile drops 1DP1g and 3DP1g (without an electric field), and 2DP1gEF and 4DP1gEF (with an electric field). Microgravity conditions: evaporation rates of sessile drops 7DP*μ*g (without an electric field) and 8DP*μ*gEF (with an electric field). The parameters of sessile drop 7DP*μ*g were used for the calculation of the analytical diffusion-limited evaporation rate without an electric field^20^. Error bars are calculated estimating the minimum and maximum evaporation rate experimentally measured.

**Fig. 5:**
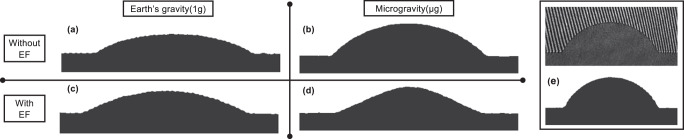
Comparison of the sessile drop interface under the effect of gravitational and electrical field forces. **a** Drop 1DP1g on the ground at *t* = 2.3 s. **b** Drop 7DP*μ*g under microgravity at *t* = 30 s. **c** Drop 2DP1gEF on the ground with an electric field at *t* = 2.4 s. **d** Drop 6DP*μ*gEF under microgravity with an electric field at *t* = 6.8 s. **e** Image from a side-view camera with interferometry lines (top) and after cleaning (bottom). The cleaned images are used to measure volume over the time (see Fig. 3).

A comparison of experimental and theoretical evaporation rates is presented in Fig. 4 for drop 7DP*μ*g under microgravity conditions at time *t* = 30 s (see Figs. 3 and 5b for a side view). The parameters for the analytical calculation are the base radius *L* = 2 mm, contact angle *θ* = 45. 6°, *D*_eff_ = 5.4 × 10^−6^ m^2^ s^−1^, *P*_sat_ = 27,268 Pa, *M*_l_ = 0.25 Kg mol^−1^, *P*_amb_ = 105,100 Pa, and *T*_amb_ = 25.36 °C. The calculated theoretical value of the diffusion-limited evaporation rate for this drop (7DP*μ*g) under microgravity conditions at time *t* = 30 s is 0.095 μL s^−1^. The experimental value for the time evolution of the sessile drop volume is calculated from post-processing the side view of the drop shape (see Fig. 5e). The experimental values under Earth and microgravity conditions without electric field are 0.198 and 0.087 μL s^−1^, respectively. This technique is more accurate in the constant contact area evaporation mode with an uncertainty maximum up to ±0.05 μL for the volume and of ±0.015 μL for the evaporation rate.

Drop shapes result from body forces equilibrium during the evaporation process. Fig. 5 shows the comparison of the sessile drop under gravity only (see Fig. 5a), microgravity only (see Fig 5b), both gravity and electric field (see Fig. 5c), and finally microgravity and electric field (see Fig. 5d). The combination of body forces results in changes of interface curvature, contact angle, and thus in the de-pining stage. Figure 5e is only intended to show the comparison between raw images from experiments (top) and clean ones (bottom) after post-processing. The cleaned images have been later used to calculate the time evolution of drop volumes reported in Fig. 3.

**Fig. 6:**
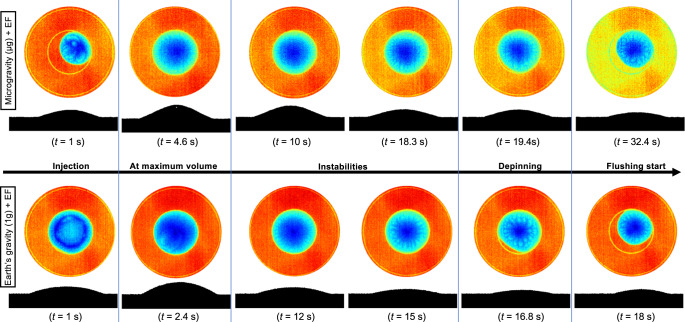
Time series of infrared images (top view) during the evaporation of liquid HFE-7100 sessile drops on a heated substrate under microgravity and Earth conditions with electric field (EF). The frames illustrate the injection, instability pattern, and de-pinning stages, respectively, for drops 6DP*μ*gEF and 4DP1gEF under microgravity (top) and Earth’s gravitational conditions (bottom) (see Table 1 and refer to the Supplementary Materials for complete movies).

To better understand the overall evaporation process, it could be interesting to address the related coupled fluid-flow problem that is induced. For that purpose, Fig. 6 displays top view IR and side-view images of drop 6DP*μ*gEF in the microgravity experiment and drop 4DP1gEF in the ground experiment subjected to an 8 kV electric field, as these were the only two drops of similar initial volume (see Fig. 3). The drop evaporation time series is divided in five sections, starting the sequence from the liquid injection to flushing. Next to the injection phase, surface temperature was almost uniform in both experiments, until thermo-capillary instabilities take place for drop 6DP*μ*gEF at *t* = 18.3 s in the microgravity experiment and drop 4DP1gEF at *t* = 12 s in the ground experiment. The pattern of thermo-capillary instabilities shows several cells coming from bottom to surface of sessile drop and then moving toward the contact line. It clearly appears that these thermo-capillary instabilities only occur once the drop volume gets below a critical value (see horizontal lines in Fig. 3 and detailed values in Table 1). It is noteworthy from Fig. 3 that these thermo-convective instabilities do not significantly modify the evaporation rates, whatever been under Earth or microgravity conditions. The last two sections of Fig. 6 display the initiation stage of de-pinning and that of flushing, respectively.

## Discussion

Owing to unrepeatable injection drop volumes, one was faced with very different initial evaporation conditions between Earth and microgravity experiments (see Fig. 3). Moreover, the time plot for drop 5DP*μ*g under microgravity conditions (see Fig. 3) exhibits some oscillations until de-pinning occurs. The detailed reasons for this strange behavior are under investigations, but the oscillations in volume may be related to higher mechanical coupling to the rocket vibrations due to its initial volume being larger than that of the other drops (see Table 1). It might also have resulted from the release of gas bubbles inside the drop during evaporation, as can be observed from the side-view images of the drop. The global evaporation rate of drop 5DP*μ*g (microgravity) is thus excluded in the subsequent analysis.

The effect of gravity on the evaporation rate clearly appears in Fig. 4: its value is roughly halved under microgravity conditions as compared to Earth conditions; this is in agreement with previous works^11,17,22^. Indeed, the average evaporation rate of the sessile drops of HFE-7100 under microgravity is 56% and 45% lower than that under Earth conditions without and with the electric field, respectively. Interestingly, the analytical diffusion-limited evaporation rate enables us to conclude that the average evaporation rate of HFE-7100 sessile drops under microgravity conditions in the absence of an electric field seems to be mainly controlled by diffusion. Furthermore, note that the average evaporation rate under Earth conditions with an electric field is 6% lower than the average rate without one, whereas the average evaporation rate under microgravity conditions with an electric field is 19% higher than the average rate without one. That is to say, the effect of an electric field on the evaporation rate of HFE-7100 is opposite under microgravity and Earth conditions, as it is for liquid water drops^22^.

Figure 5 shows side views of the sessile drops under the four considered conditions. For a fair comparison, compare Fig. 5a with Fig. 5c and Fig. 5d with Fig. 6 (drop 4DP1gEF), as these drops were of comparable volumes (see Table 1). Also, it is noteworthy that no drop in microgravity without an electric field had a lower initial injected volume (see Fig. 3b). Therefore, the minimum volume for drop 7DP*μ*g under microgravity at *t* = 30 s can be used for comparison of the interface. The interface shape of the sessile drops resulted from body and surface forces acting on them. As it clearly appears in Fig. 5a, b, the shape of a sessile drop under microgravity is exactly spherical in comparison to that in Fig. 5a. In contrast, sessile drops exhibit clear cone formation under microgravity conditions with an electric field (see Figs. 5d, c and 6). Along with the influence on the interface (see Fig. 5), which is in agreement with other experiments^10,12,18,22,23^, the de-pinning process is also associated with the gravitational and electrical forces individually or in combination. Based on these comparisons, we can see the correlation between the body and surface force conditions and the volumes (see Fig. 3) and contact angles (contact angles were measured by using the ImageJ software plugin known as DropSnake, which is based on B-spline snakes (active contours)) during de-pinning irrespective of the shape of the sessile drop interface shape. The fact that de-pinning is anticipated in the presence of an electric field can be attributed to the fact that the radial electric force is directed inwards, causing striction of the interface^12^. Accordingly, the order of de-pinning based on the volume and contact angle and body and surface force conditions was as follows: drop 6DP*μ*gEF with an electric field (under microgravity conditions) at volume = 1.83 μL and contact angle *θ* = 18.7° de-pinned at the highest volume and contact angle and did so earlier than drop 2DP1gEF with an electric field (under Earth conditions), which de-pinned at volume = 0.81 μL and contact angle *θ* = 14.6°, and drop 4DP1gEF with electric field (under Earth conditions), which de-pinned at volume 0.76 μL and contact angle *θ* = 13.4°. Drop 3DP1g without an electric field (under Earth conditions) at volume = 0.43 μL and contact angle *θ* = 8.6° and drop 1DP1g without an electric field (under Earth conditions) at volume = 0.43 μL and contact angle *θ* = 7.6° de-pinned with the smallest volumes and contact angles. According to the above correlations, it can be predicted that for sessile drops 5DP*μ*g and 7DP*μ*g (under microgravity without an electric field), the volume (and contact angle) should have been either equal to or higher than the volume (and contact angle) of drops 2DP1gEF and 2DP1gEF (under Earth conditions with an electric field) at de-pinning. The influence of the substrate grooves in the de-pinning dynamics could itself be a subject of investigation^24^.

The IR images in Fig. 6 reveal some characteristic patterns associated with the thermo-capillary instabilities that occurred for drop 6DP*μ*gEF in microgravity conditions at time *t* = 18.3 s, which corresponds to a volume of 2.01 μL, calculated using the side-view image (refer Fig. 6) in which the maximum sessile drop height is 0.41 mm. The thermo-capillary instabilities first appeared near the periphery of the sessile drop during evaporation and before de-pinning, and they remained visible up to complete evaporation (see Fig. 6). In the ground experiment, however, there were instability patterns for drop 4DP1gEF stating at time *t* = 15 s and volume = 1.67 μL (maximum interface height of 0.24 mm); the patterns began to appear at time *t* = 12 s and volume = 1.10 μL. Similarly, instability patterns appeared in all the sessile drops in the ground reference experiment (see Fig. 3), for which volume and time are reported in Fig. 3. The thermo-capillary instabilities appeared as soon as the maximum drop height was below a critical value of approximately between 0.2 and 0.3 mm for Earth conditions and around 0.4 mm for the microgravity conditions, which is associated with thermo-capillary instabilities referred to as Marangoni instabilities. Interestingly, the above critical thickness for HFE-7100 under Earth conditions fully agrees with Chauvet et al.^25^. Therefore, as the injected volume of most of the microgravity drops exceeded that of the drops in the Earth reference experiment, longer evaporation times would have been required for the former to reach the critical height at which thermo-capillary instabilities are observed. As a result, flushing of the largest microgravity sessile drops was unfortunately performed before instability patterns could be observed.

In conclusion, it is worth mentioning that it was very difficult to carry out repeatable injection of prescribed sessile drop volume both under Earth and microgravity conditions. The exact reasons for the formation of oversized sessile drops under microgravity conditions are still under investigation. In future microgravity experiments, it would therefore be preferable to perform sessile drop volume injection with real-time feedback control. Under our experimental conditions, the results provide evidence for the effect of microgravity conditions on the sessile drop evaporation rate, indicating that the rate under microgravity conditions is nearly half that under Earth conditions for HFE-7100. Furthermore, the effect of an electric field on the evaporation rate is opposite under microgravity and Earth conditions. The experimental results also demonstrate the relationship between thermo-capillary instabilities and the measured critical height of the sessile drop interface. For temperature differences between substrate and ambient in the range of 2–3 °C with a base diameter of 4 mm, the measured critical height for the appearance of thermo-capillary instabilities is approximately between 0.2 and 0.3 mm for Earth conditions and around 0.4 mm for the microgravity conditions for HFE-7100. It is also noteworthy that meanwhile they strongly change the fluid-flow structure in the sessile drop, these thermo-capillary instabilities do not significantly influence the evaporation rates. Through the application of different combinations of volumetric forces (an electric field and gravity), we also demonstrate the role of gravity on the shape of the sessile drop interface and its influence on the de-pinning of sessile drops. To concrete the above evidence, module will re-fly again (as a baseline, in 2022). One of the main objectives of the reflight is to better control the actual injected volumes so as to ensure a better data comparison among the different testing conditions.

## Methods

### Fluid property measurements

In all cases, the liquid used was 99.9% pure HFE-7100 (3M^TM^ Novec^TM^ 7100 Engineered Fluid, a hydrofluoroether also known as methoxy-nonafluorobutane (C4F9OCH3)). It was chosen for its volatility, semi-transparency in the IR wavelengths, perfect wetting, non-toxicity, and being non-flammable. For more on the properties of HFE-7100, please refer to https://multimedia.3m.com/mws/media/199818O/3m-novec-7100-engineered-fluid.pdf and https://multimedia.3m.com/mws/media/569860O/3mtm-thermal-management-fluids-for-military-aerospace-apps.pdf.

### Hardware description

The ARLES experiment module was designed and manufactured by the SSC under the guidance of the ESA and Science team proposition based on the required scientific objectives. The overall design of the experiment module is subdivided into two parts (see Fig. 1a): the main evaporation cell (MEC), which is for single-drop experiment systems and the multi-drop cell, which is for multi-drop experiment systems to be executed in parallel. For safety reasons, a neutral gas nitrogen (N_2_) atmosphere was used.

#### Main evaporation cell

The chamber volume of the main evaporation cell (MEC) is 4 l. It is sized to maintain a low vapor concentration throughout the whole experiment even if the N_2_ evacuation fails during the flight. The cell thickness was chosen to withstand the expected pressure differences during the filling and emptying of the gas (N_2_). Figures 1 and 2 shows the main cell used to perform sessile drop evaporation of a pure fluid on a heated substrate with and without an electric field. The substrate was a thin single-crystal silicon wafer coated with a 50 nm-thick platinum layer, whose surface roughness was less than 1 micron RMS, deposited by atomic layer deposition. The substrate had 50 × 50 μm grooves with 4 ± 0.1 mm in diameter to force the pinning of a sessile drop with a diameter of 4 mm (see Fig. 1d). The central hole for the fluid injection was 0.7 mm in diameter. The substrates were manufactured at MICAS TU Leuwen. An IR camera was mounted on the lid of the main evaporation cell, where a ZnSe window served as the passage for IR wavelengths. The interferometry camera observed the single-drop evaporation process through the side observation windows of the MEC.

#### Multi-drop cell

The multi-drop experiment system is for the analysis of different fluids with nanoparticle suspensions, and the related pattern formation on the substrates after the evaporation process, and its consequent functionalisation. As such, it is not part of our analysis.

#### Heat flux, temperature, and pressure measurements

Two T-type thermocouples monitored the substrate temperature. One thermocouple was placed close to the center hole and the other one close to the edge of the substrate. These sensors were incorporated in the heat flux sensor by the CAPTEC manufacturer. The heat flux sensor with integrated thermocouples determined the heat flux to the drops and substrate temperature with a sensitivity of 2 μV W^−1^ m^2^). Along with the substrate temperature, we used a set of PT-100 sensors to monitor the cell wall temperature and the ambient temperature inside the MEC (see Fig. 1 for the position). The temperature measurement rate was 30.4 Hz, with an uncertainty of ±2.1 K from the true temperature in the worst case. The passband of the filter was 4.56 Hz. A dedicated *μ*-TC interface board performed the readout of the heat flux sensor and the thermocouples. The pressure sensor measured pressure in the range of 0–1.6 bar with an accuracy of ±0.2% inside the MEC throughout the experiment.

#### Heater

The heaters were custom made and manufactured by NEL Technologies Ltd. They are capton patch heaters with an etched resistive pattern. For the MEC, the heater was designed to provide 5 W of uniform heating power at 24 V. The heater was driven from 24 V pulse width modulation (PWM).

#### Electrode

The positive high voltage potential was connected to a conical shape electrode, which was located above the substrate, concentric with the substrate grooves and the drop injection inlet hole, at a distance of 6 mm from the substrate (see Fig. 2). On the other hand, the substrate is connected to negative voltage potential.

### Image acquisition and analysis

To perform fluid-flow visualization of the drops, high-resolution IR images were captured by a commercial off-the-shelf bolometric (non-cooled) Xenics, Gobi 640 camera. The images are 640 × 480 pixels (H × V) with a noise-equivalent temperature difference of 50 mK at 30 °C and an IR wavelength region of 8–12 μm. The images were recorded via an IR optical path consisting of an AR-coated ZnSe window (75 × 6 mm). The depth-of-field of the IR camera with an image pixel density of 17 μm is 0.7 mm at 9 cycles mm^−1^. To visualize and track the evolution of the interface of the sessile drops, we used images from the side-view camera of the interferometer. The interferometer images have a field-of-view of 15 × 15 mm with an image pixel density of 10.78 μm pixels^−1^ (11.2 μm pixels^−1^ for microgravity conditions). The interferometry fringes were removed from the raw images with the help of the ImageJ software. The cleaned images without fringes were used for the analysis (see Fig. 5e). The image acquisition rate for all the images was 25 Hz.

### Experimental procedure

The SubOrbital Express rocket (MASER 14) launch took place successfully on Monday, 24 June 2019, from the Esrange Space Center in northern Sweden. The atmospheric replacement was executed 60 s after the launch by feeding in the N_2_ while the experimental cells were connected to an exhaust port in the outer structure. At the start of the microgravity phase (100 km level) *t* = 70.4 s, the experiment liquid was injected to create the first drop of HFE-7100 upon the heated substrate. After a delay corresponding to the estimated drop evaporation time, the atmosphere in the chamber was flushed. After the flushing sequence, another drop was injected, and the evaporation cycle with diagnostics was repeated. The outside pressure was 0 bar during the microgravity period. At the bottom of the ARLES experiment module is an N_2_ pressure vessel for flushing the single-drop cell after each consecutive drop. Flushing was performed to prevent the evaporated liquid from condensing in the experiment cell. The ground test experiment were executed in the same way as during the flight. The only difference was the membrane vacuum pump, which was connected to the exhaust of the module.

### Reporting summary

Further information on research design is available in the Nature Research Reporting Summary linked to this article.

## Supplementary information

SUPPLEMENTAL MATERIAL

The evaporation of liquid HFE-7100 sessile drops on a heated substrate under microgravity and Earth conditions with electric field (EF)

Reporting Summary Checklist

## Data Availability

The data collected during this study is available from the corresponding authors upon reasonable request.
