# Supplementary material for: Sessile volatile drop evaporation under microgravity
Source: NPJ Microgravity. 2020 Dec 11;6:37. doi: 10.1038/s41526-020-00128-2 (PMC7733520; doi:10.1038/s41526-020-00128-2)
Supplement: Supplementary file 1 — SUPPLEMENTAL MATERIAL [file 41526_2020_128_MOESM1_ESM.pdf]

Supplementary Movie 1. The movie shows the comparisons for the infrared (top) and side view (bottom) during the evaporation of liquid HFE-7100 sessile drops on a heated substrate under microgravity and Earth conditions with electric field (EF), which is corresponding to Figure 6. The comparisons show the injection, instability pattern, de-pinning, and in the end flushing stage for drops 6DPμgEF and 4DP1gEF under microgravity (left) and Earth's gravitational conditions (right).
